# MitoMiner v3.1, an update on the mitochondrial proteomics database

**DOI:** 10.1093/nar/gkv1001

**Published:** 2015-10-01

**Authors:** Anthony C. Smith, Alan J. Robinson

**Affiliations:** MRC Mitochondrial Biology Unit, Wellcome Trust/MRC Building, Cambridge Biomedical Campus, Hills Road, Cambridge, CB2 0XY, UK

## Abstract

Mitochondrial proteins remain the subject of intense research interest due to their implication in an increasing number of different conditions including mitochondrial and metabolic disease, cancer, and neuromuscular degenerative and age-related disorders. However, the mitochondrial proteome has yet to be accurately and comprehensively defined, despite many studies. To support mitochondrial research, we developed MitoMiner (http://mitominer.mrc-mbu.cam.ac.uk), a freely accessible mitochondrial proteomics database. MitoMiner integrates different types of subcellular localisation evidence with protein information from public resources, and so provides a comprehensive central resource for data on mitochondrial protein localisation. Here we report important updates to the database including the addition of subcellular immunofluorescent staining results from the Human Protein Atlas, computational predictions of mitochondrial targeting sequences, and additional large-scale mass-spectrometry and GFP tagging data sets. This evidence is shared across the 12 species in MitoMiner (now including *Schizosaccharomyces pombe*) by homology mapping. MitoMiner provides multiple ways of querying the data including simple text searches, predefined queries and custom queries created using the interactive QueryBuilder. For remote programmatic access, API's are available for several programming languages. This combination of data and flexible querying makes MitoMiner a unique platform to investigate mitochondrial proteins, with application in mitochondrial research and prioritising candidate mitochondrial disease genes.

## INTRODUCTION

Mitochondria are involved in a diverse range of cellular processes including metabolism, energy production, signalling, cell growth and apoptosis. They are mobile organelles constantly fusing, dividing and replicating, and have tissue specific roles such as ammonia detoxification in liver. It is therefore unsurprising these organelles are associated with a wide spectrum of metabolic, degenerative and age-related human diseases as well as cancer. This has generated considerable interest in mitochondria from a wide range of researchers. However, much of the mitochondrial proteome has yet to be conclusively identified which hinders investigations into the role of the organelle. Many different approaches have been used to address this problem, but each has limitations and no single technique provides full coverage of the mitochondrial proteome. Numerous mass spectrometry experiments have identified proteins in purified fractions of mitochondria, but a proportion of these proteins are cellular contaminants, and the results are limited to identifying proteins expressed in the tissue type examined. Further, it is challenging to extract and cross-reference results from these studies, as the data are usually published as supplementary tables with varying identifiers. A different approach uses GFP tagging to identify mitochondrial proteins. However, the tag can interfere with translocation of the protein into mitochondria. In addition, the approach is time-consuming and technically challenging in mammals and so many of these data sets originate from yeast, although these have functionally distinct mitochondria compared to higher eukaryotes. Computational methods have focussed on predicting subcellular targeting motifs in the N-termini of protein sequences (1–3). However, many known mitochondrial proteins lack a targeting sequence whereas many other proteins are predicted to have one but are experimentally found not to localise to the organelle. The Gene Ontology provides literature-based annotation of proteins, including subcellular localisation (4). However, this is an indivisible combination of annotation for well-characterised proteins whose mitochondrial localisation has been conclusively determined, and annotation derived from (often only single) large-scale localisation studies that include many false positives. The most recent effort has been from the Human Protein Atlas (5), which used antibodies to immunofluorescently stain proteins and localise them by microscopy. But this approach may suffer from cross reactivity and staining failures. Thus cross-referencing between these different evidence types would be useful to independently verify candidates and reduce false positive rates, and was the premise for the first version of MitoMiner (6), which then only included mass spectrometry and GFP tagging data from 33 studies with Gene Ontology annotation. We have now updated MitoMiner to include the new localisation evidence from the Human Protein Atlas, mitochondrial targeting sequence predictions and have expanded the number of experimental studies to 58. Homology information from HomoloGene (7) allows this evidence to be shared across the 12 species in MitoMiner (*Homo sapiens*, *Mus musculus*, *Rattus norvegicus*, *Bos taurus*, *Drosophila melanogaster*, *Arabidopsis thaliana*, *Saccharomyces cerevisiae*, *Schizosaccharomyces pombe*, *Plasmodium falciparum*, *Neurospora crassa*, *Tetrahymena thermophila* and *Giardia lamblia*).

MitoMiner has a complementary role of giving a biological context for candidate mitochondrial proteins by integrating information from other public resources. This provides a useful and flexible starting point for many analyses, such as assessing and prioritising candidates generated from ‘omics data sets or exome sequencing of mitochondrial disease patients. This information includes annotation from UniProt (8), and the Gene Ontology (9), metabolic pathway data from KEGG (10), disease information from OMIM (11) and (new to latest version) tissue and cancer expression from the Human Protein Atlas (5) and InterPro protein domain information (12). To query these data, MitoMiner provides a powerful and flexible user interface, allowing everything from simple text searches to complicated queries with multiple constraints spanning any of the included data types, (see previous publications for a detailed description (6,13)). Users can also run queries on uploaded lists of proteins, or use a pre-existing list such as the widely-respected MitoCarta inventory of mitochondrial proteins (14).

## SOFTWARE IMPLEMENTATION AND DATA IMPORT

To minimise development time and reduce legacy issues, MitoMiner was built using the InterMine open source data warehouse system, updated to version 1.2.2 (15). The InterMine core model is the basis for the database structure and describes types of biological data including genes, proteins, publications and hierarchical gene ontology terms. To model data types specific to MitoMiner—such as mass spectrometry and GFP tagging data sets, metabolic pathway data and homology mappings—bespoke tables were created that extend the database structure. Data were imported by using either InterMine-provided data loaders, or custom Perl scripts to convert raw data files to InterMine compatible XML data files. These scripts were designed so data updates require minimal manual intervention and so ease database maintenance. The MitoMiner data sources are updated on a 9–12 month basis.

## UPDATES TO DATA SOURCES

### Addition of new mass-spectrometry and GFP data sets

Since the last publication (13) we have increased the number of large-scale mass spectrometry and GFP tagging studies in MitoMiner from 46 to 58 (16–27). Every data entry in MitoMiner has full provenance of its originating study and for mass spectrometry includes the experimental techniques used for purification, separation and identification, to show how the authors reduced contaminants. All entries of existing data sets were remapped to UniProt to remove obsolete and redundant UniProt protein identifiers. The total number of data entries in MitoMiner by species is shown in Table 1.

**Table 1.** Summary of mitochondrial proteomics studies in MitoMiner

Species	Number of publications	Number of data entries^a^		Number of genes with experimental evidence^b^
		Mass spectrometry	GFP	
*H. sapiens*	15	4903	144	1839
*M. musculus*	12	17577	52	3076
*B. taurus*	1	28	0	30
*R. norvegicus*	9	3398	0	1836
*D. melanogaster*	1	43	0	42
*S. cerevisiae*	11	3193	1257	1291
*S. pombe*	1	0	430	432
*A. thaliana*	5	953	0	483
*N. crassa*	1	290	0	232
*T. thermophila*	1	310	0	294
*G. lamblia*	1	993	0	641

^a^The number of unique data entries from mass spectrometry or GFP tagging mitochondrial localization studies.

^b^The number of unique Ensembl gene identifiers. Does not include mitochondrial evidence from homologs.

### Addition of mitochondrial targeting sequence predictions

Many programs have been developed to predict subcellular targeting motifs in protein sequences. All these programs have web services to scan individual sequences, but with a large number of candidates this is cumbersome and hinders comparison with other localisation evidence. Therefore, in this update MitoMiner now includes the results from three popular mitochondrial target sequence prediction programs: iPSORT (1), TargetP (2) and MITOPROT (3). For each program, MitoMiner stores the prediction score for every protein in the proteome of the 12 species included, which allows different score thresholds for each program to be used in queries. The number of proteins predicted to have a mitochondrial targeting sequence, by species is shown in Table 2.

**Table 2.** Summary of mitochondrial targeting sequence predictions in different proteomes

Species	Number of genes encoding proteins with a predicted mitochondrial targeting sequence			Total
	iPSORT^a^	MitoProt^b^	TargetP^b^	
*H. sapiens*	3940	1886	387	4716
*M. musculus*	3052	1526	363	3679
*B. taurus*	2312	1235	267	2911
*R. norvegicus*	2617	1350	297	3220
*D. melanogaster*	1654	916	187	1990
*S. cerevisiae*	991	585	120	1182
*S. pombe*	684	389	81	822
*A. thaliana*	4871	2281	927	6323
*N. crassa*	1039	571	268	1161
*T. thermophila*	1678	827	36	2133
*G. lamblia*	909	282	60	1023

^a^With a score of 1.0 (scoring is binary).

^b^With a score equal to or greater than 0.9.

### Addition of data from the human protein atlas

**Figure 1.**
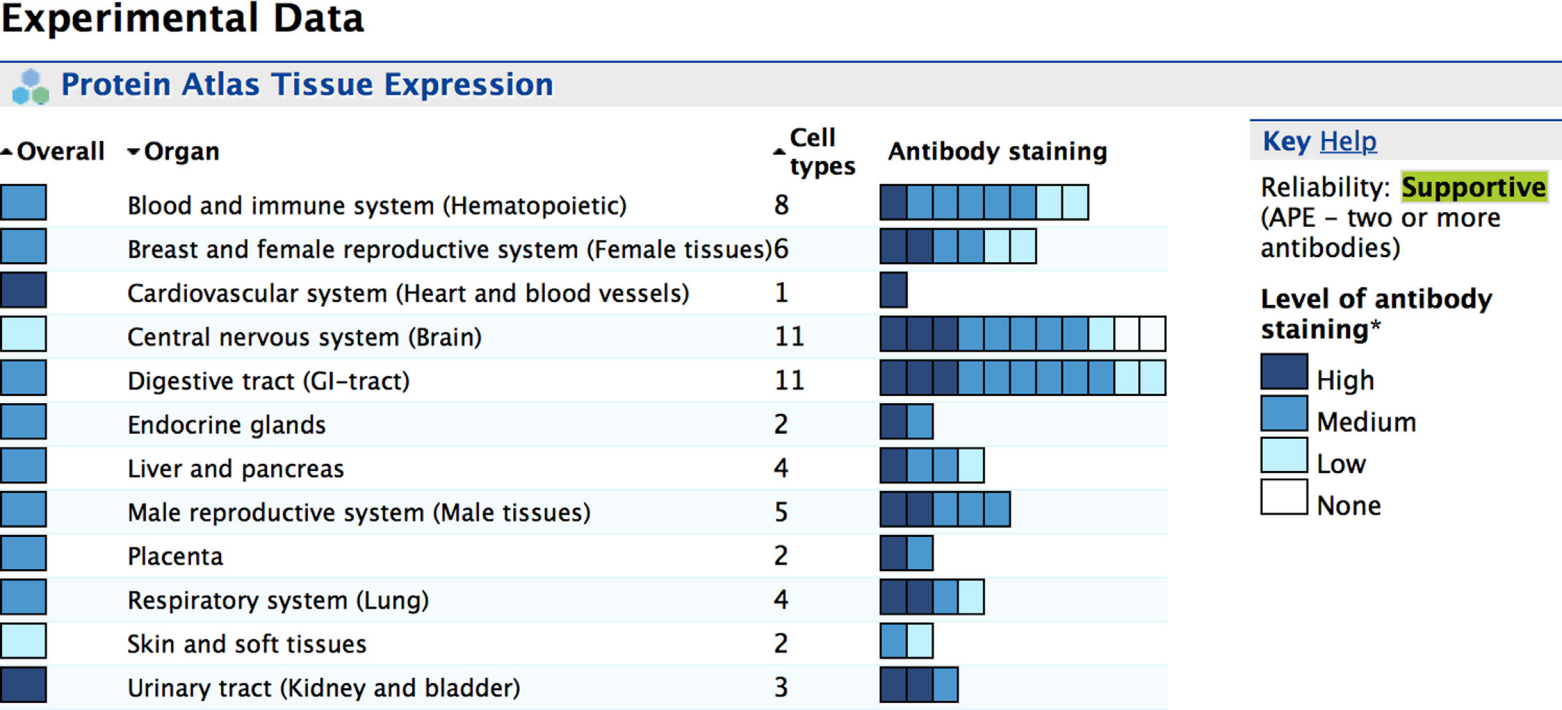
Graphical summary of Human Protein Atlas tissue expression data for a mitochondrial protein in MitoMiner.

The most important new type of large-scale subcellular localisation data comes from immunofluorescent staining and microscopy conducted by the Human Protein Atlas (HPA) (5). For each protein with HPA data we incorporated the original Ensembl gene identifier, main subcellular location reported, any other subcellular locations, expression type (whether localisation has been confirmed with multiple antibodies) and reliability (does this the location agree with UniProt annotation). To provide more biological context for protein entries, we also incorporated the HPA immunohistochemical expression results from 59 different tissues and 20 cancer types. For tissue expression we included tissue name, tissue group, cell type, expression type, expression level and reliability. To aid interpreting these data we used an InterMine graphical summary to provide the results in an easily understandable format (Figure 1). For cancer expression we included the original Ensembl gene identifier, tumour type, number of patient samples with a particular level of expression (strong, moderate, weak or negative) and expression type.

### Other improvements

To improve the searchability of MitoMiner for gene-based queries and analyses (such as in identifying mitochondrial genes amongst variants found in exome sequencing), we expanded gene information to include HUGO gene symbol, Ensembl identifier, Ensembl gene description, chromosome, NCBI gene identifier and model organism specific gene identifiers (e.g. from Mouse Genome Database, Rat Genome Database and *Saccharomcyes* Genome Database). To improve metabolic analyses for systems biology applications, KEGG reaction entries were expanded to include the reaction's estimated change in Gibbs free energy (ΔG) (28), the reaction directionality defined by KEGG, and the reaction equation using KEGG compound identifiers. Protein entries now include InterPro domain information (29) enabling queries for subsets of (novel) mitochondrial proteins with particular functions—e.g. RNA binding. Remote programmatic access via the Application Programming Interface (API) was improved with the updated version of the InterMine software and includes client libraries for Ruby in addition to Perl, Python and Java. Lastly the documentation, tutorials and user guides have been extensively updated.

## AVAILABILITY

MitoMiner is freely available at the Medical Research Council Mitochondrial Biology Unit website (http://mitominer.mrc-mbu.cam.ac.uk/). The main website is accompanied with a full set of support pages including FAQ's, user guides, examples and tutorials (http://mitominer.mrc-mbu.cam.ac.uk/support).
